# Deep Learning Framework for Automated MRI Planimetry in Multiple Sclerosis

**DOI:** 10.1155/ijbi/4456355

**Published:** 2026-03-06

**Authors:** Stephanie Mangesius, Daniela Schiefeneder, Matthias Schwab, Markus Tiefenthaler, Florian Deisenhammer, Markus Haltmeier, Elke R. Gizewski

**Affiliations:** ^1^ Department of Radiology, Medical University of Innsbruck, Innsbruck, Austria, i-med.ac.at; ^2^ Neuroimaging Core Facility, Medical University of Innsbruck, Innsbruck, Austria, i-med.ac.at; ^3^ Department of Mathematics, University of Innsbruck, Innsbruck, Austria, uibk.ac.at; ^4^ Department of Neurology, Medical University of Innsbruck, Innsbruck, Austria, i-med.ac.at

**Keywords:** deep learning, midsagittal plane detection, MRI planimetry, multiple sclerosis

## Abstract

Brain volume changes and infratentorial involvement are key predictors of disability in multiple sclerosis (MS) and can be assessed using magnetic resonance imaging (MRI) planimetry. Although MRI planimetry is less susceptible to methodological and patient‐related confounders than volumetry, it currently depends on manual measurements by unblinded experts, an approach that is time‐consuming and vulnerable to bias. In this study, we present a fully automated deep learning framework for deriving brainstem planimetric measurements from MRI. The pipeline integrates an automated midsagittal plane (MSP) detection algorithm with a convolutional neural network trained to perform the segmentations required for planimetry. The automated method shows strong agreement with manual measurements and remains robust across scanners and acquisition protocols. These findings suggest that the proposed framework enables reliable, reproducible, and scalable MRI planimetry, supporting objective assessment of disease progression and treatment response in patients with MS.

## 1. Introduction

MS is a chronic, neuroinflammatory, and neurodegenerative disorder of the central nervous system [1, 2]. Early neuronal and axonal loss leads to measurable brain volume changes [3–5], and progressive atrophy has been proposed as an MRI‐based predictor of irreversible disability [6, 7]. Infratentorial involvement, in particular, is strongly associated with poorer clinical outcomes [8]. While MRI is a powerful tool for monitoring these structural changes, conventional imaging often provides limited or subjective information directly relevant to disease progression. This highlights the need for quantitative and reproducible imaging biomarkers to support early diagnosis, accurate prognosis, and informed clinical decision‐making.

MRI volumetry can improve diagnostic accuracy but is often limited by scanner variability, availability, and complex processing requirements. In contrast, MRI planimetry provides robust and readily obtainable metrics that can be evaluated using standard imaging software. Several studies have demonstrated the diagnostic utility of brainstem planimetric measures, including midbrain and pontine areas and diameters, as well as the magnetic resonance parkinsonism index (MRPI), for differentiating patients with neurodegenerative disorders [9, 10].

Planimetry is typically performed on the midsagittal plane (MSP), a single well‐defined slice of the MRI volume. Although manual determination and measurement are straightforward for radiologists, the process is time‐consuming, rater‐dependent, and difficult to standardize across sites [11]. Fully automated planimetric workflows have not yet been established. These limitations underscore the need for automated, reproducible methods for both MSP extraction and planimetric measurement, motivating the development of a deep learning–based frameworks for automated planimetry.

The primary aim of this study is to develop and evaluate a deep learning framework for automated and standardized MRI planimetry. The framework focuses on brainstem structures that are particularly susceptible to atrophy and may serve as predictors of disease progression or treatment response. The proposed approach comprises the following:

1. Automated extraction of the MSP from 3D MRI volumes.
2. Convolutional neural network (CNN)–based segmentation of the midbrain and pons.
3. Planimetric measurement of areas and diameters from the segmented MSP.
4. Use of a weighted entropy loss function to address class imbalance during network training.

Evaluation was performed on a cohort of MS patients across multiple subtypes, with automated results compared against manual planimetric measurements by medical experts, which is considered a gold standard reference.

While the novelty of this work does not lie in the development of a new CNN architecture, the innovation resides in the first application of CNNs to automated brainstem MRI planimetry, combined with task‐specific loss adjustments. The framework demonstrates robust, reproducible, and scalable performance, and the pipeline can accommodate more advanced network architectures in future work without modification to its overall design.

Table 1 summarizes abbreviations used in this work.

**Table 1:** Abbreviations used.

Abbreviation	Definition
MRI	Magnetic resonance imaging
MS	Multiple sclerosis
MSP	Midsagittal plane
EDSS	Expanded Disability Status Scale
CNN	Convolutional neural network
ROI	Region of interest
SNR	Signal‐to‐noise ratio
DICE	Sørensen–Dice coefficient
AD	Area difference
RAAD	Relative absolute area difference
ADD	Absolute diameter difference
MPRAGE	Magnetization‐prepared rapid gradient echo
HAUS	Hausdorff distance
RADD	Relative absolute diameter difference
SD	Standard deviation

## 2. Materials and Methods

### 2.1. Background

MRI planimetry has been applied to neurodegenerative and neuroinflammatory disorders to quantify midbrain and pontine structures, supporting early diagnosis and disease monitoring. Previous methods rely on manual or semiautomatic segmentation, which is labor‐intensive and subject to interrater variability [12–19]. While automated MSP detection and brainstem segmentation methods have been proposed [20, 21], these approaches often suffer from limited generalizability, small datasets, or a lack of integration with downstream clinical applications.

Deep learning–based segmentation techniques have demonstrated high accuracy in neuroimaging tasks [22–26]; however, fully automated MRI planimetry remains unexplored. This study addresses this gap by presenting an end‐to‐end framework for MSP detection, segmentation, and planimetric measurement, enabling reproducible assessment across a heterogeneous patient cohort. To the best of our knowledge, no other automated MRI planimetry pipeline currently exists, and we benchmark our method against manual planimetric measurements, considered the gold standard.

### 2.2. Dataset

High‐resolution MRI scans were acquired using four 1.5 T and two 3 T Siemens scanners with a standard multichannel head coil. T1‐weighted 3D magnetization‐prepared rapid gradient echo (MPRAGE) sequences were obtained for all participants at the Neuroimaging Core Facility. Previous studies indicate that MRI planimetry produces consistent results across 1.5 and 3 T scanners [27]. Clinical assessments included demographic data, disease onset, and the Expanded Disability Status Scale (EDSS); see Table 2.

**Table 2:** Demographics and MRI acquisition details for all cohorts.

	Training	Validation	Test	Healthy
Patients (MRI)	56 (136)	9 (18)	22 (54)	19 (19)
No. of MRIs per patient	2.43 ± 1.56	2 ± 1.49	2.45 ± 1.92	1
Sex (m/f)	22/34	1/8	9/13	5/14
Age at MRI	46.4 ± 9.71	44.94 ± 6.36	43.02 ± 8.54	37.32 ± 13.15
Disease duration	16.34 ± 9.28	15.06 ± 8.55	15.35 ± 9.7	0
EDSS score	4.9 ± 1.73	4.19 ± 2.15	4.76 ± 1.86	0
Contrast agent	29 (21.32%)	1 (5.56%)	13 (24.07%)	0
Scanner 1.5/3 T	92/44	9/9	45/9	—

The dataset comprises 227 MRI volumes from 106 participants: 87 patients with MS (208 MRI scans) and 19 healthy controls (19 MRI scans). MS patients were randomly assigned to training (136 MRI scans), validation (18 MRI scans), and test (54 MRI scans) sets. Healthy controls were used exclusively for an additional test set (19 MRI scans). The split was performed at the patient level, as scan‐level splitting can introduce bias and data leakage if data from the same patient appear in both training and test sets, potentially inflating performance metrics.

All procedures were approved by the local Ethics Committee of the Medical University of Innsbruck. Written informed consent was waived for MS patients due to the retrospective study design (Ethics 1267/2019). Prospective healthy controls provided written informed consent (AN 5100 325/4.19 384/5.3 4213a).

### 2.3. MRI Planimetry

Planimetric measurements are parameters derived from MRI scans restricted to the MSP. Several of these measures have been proposed to quantify atrophy in specific brainstem structures and serve as disease‐specific markers. Previous studies have demonstrated their high diagnostic utility [9, 28, 29]. In this study, we focus on four specific parameters: midbrain diameter, midbrain area, pontine area, and pontine diameter.

The workflow for obtaining brainstem‐derived measures involves determining the MSP, identifying the midbrain and pons regions, and computing the area and diameter of these regions. While previous studies performed the first two steps manually, the aim of the present study is to replace them with fully automated algorithms.

### 2.4. Automated MSP Computation

The MSP was automatically extracted from each 3D MRI scan using a self‐developed algorithm that combines the 2D Radon transform with symmetry metrics [30–33]. The MSP, which separates the brain into left and right hemispheres, is defined by its roll angle ((*z*, *x*)‐plane), yaw angle ((*y*, *x*)‐plane), and its perpendicular distance from the origin.

The algorithm proceeds as follows (compare with Figure 1). First, an axial slice *F* in the upper brain is selected, and the center of mass of the thresholded binary image is computed. We then apply the Radon transform, which computes the average of *$* *F* *$* along lines characterized by normal direction *θ* and signed perpendicular distance *s*. The values *θ* ^∗^ (yaw angle) and *s* ^∗^ (perpendicular distance) are identified as the point in the Radon transform with minimal intensity value, corresponding to the intersection of the MSP with the selected axial plane. All axial slices are subsequently rotated by −*θ* ^∗^ to align them with the coronal plane.

Figure 1Workflow of MSP detection. (a) Axial slice with center of mass, (b) Radon transform with the minimal value highlighted, and (c) axial slice showing the line corresponding to this minimal value. (d) Coronal slice with the computed point, (e) intermediate alignment, and (f) final MSP.

**Figure:**
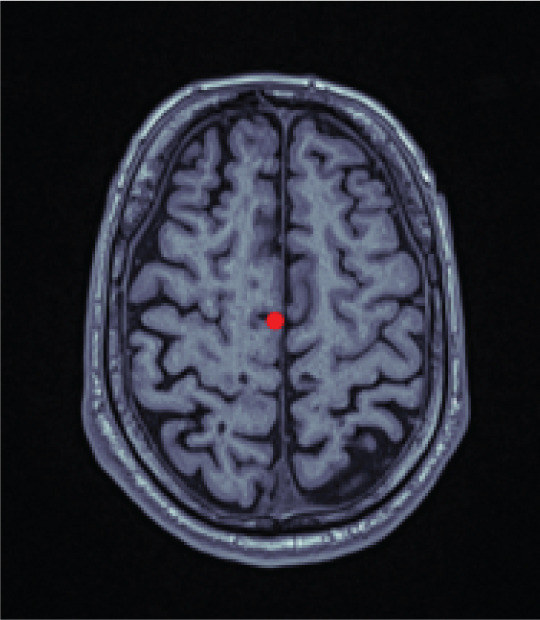
(a)

**Figure:**
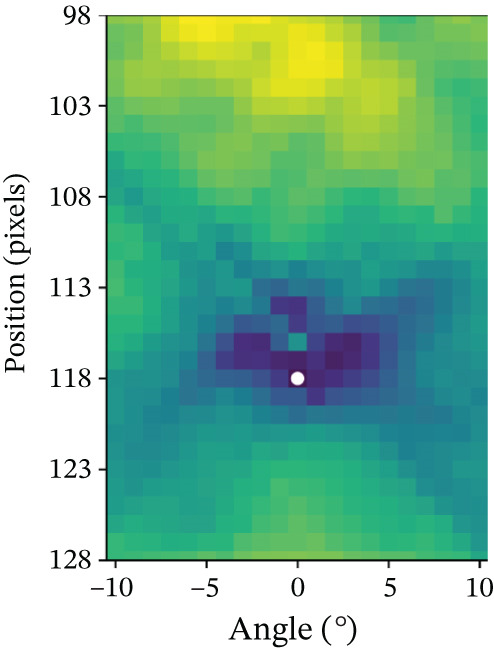
(b)

**Figure:**
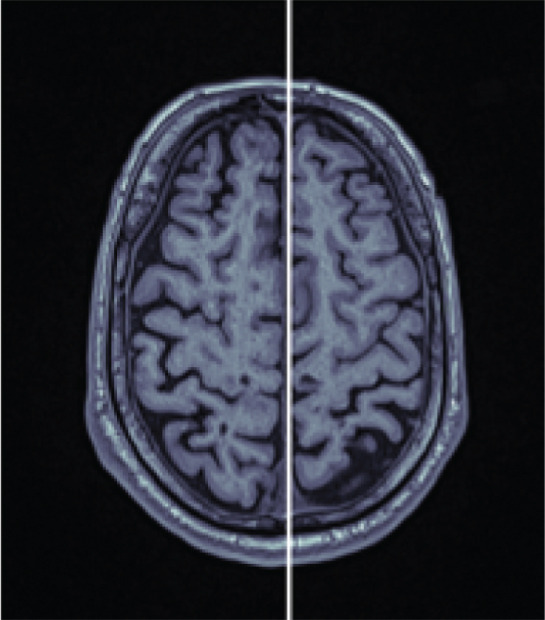
(c)

**Figure:**
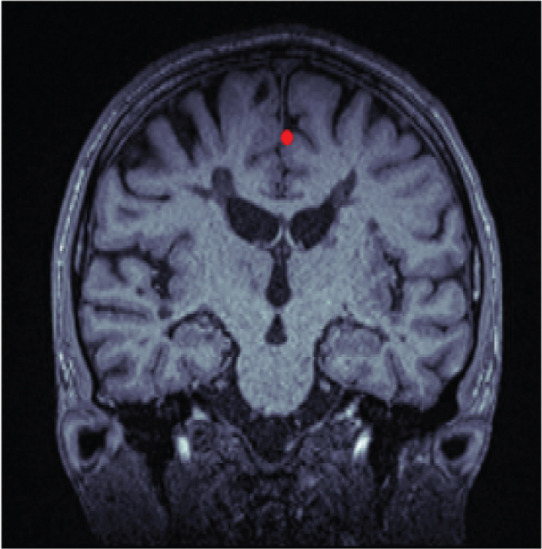
(d)

**Figure:**
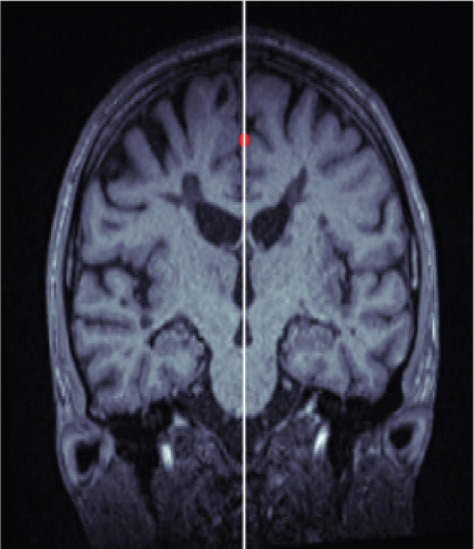
(e)

**Figure:**
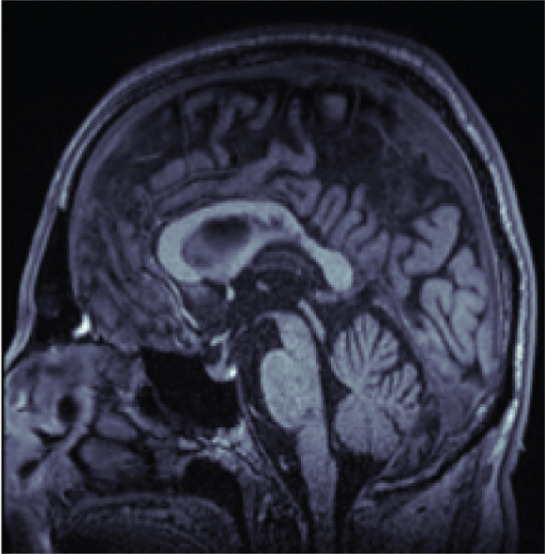
(f)

Next, the central coronal slice is rotated over candidate roll angles *ϕ* ∈ (−10, 10), and the angle that maximizes vertical symmetry is selected as the roll angle. All coronal slices are rotated accordingly. The MSP is then defined as the sagittal slice intersecting the computed line in each coronal slice. Median values of yaw, roll, and perpendicular distance across slices are used to improve robustness.

Finally, all MSP images are resampled to 176 × 256 pixels, and a 128 × 128 ROI consistently containing the midbrain and pons is extracted.

### 2.5. Manual Planimetric Measurements

Manual measurements served as the reference standard for training and validating the automated method. The procedure, following established protocols [9], was independently applied in this study and is described below. Segmentation and quantitative extraction were performed in MATLAB.

**Figure 2:**
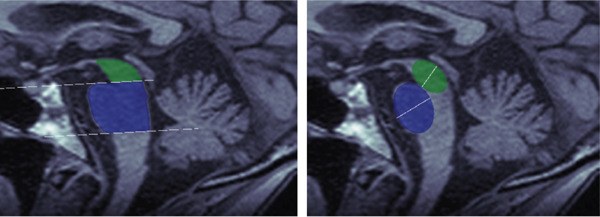
Manual planimetric measurements of midbrain (green) and pons (blue).

Midbrain and pontine areas were derived by first defining two reference lines: one extending from the inferior edge of the quadrigeminal plate (tectum) to the superior pontine notch and a second parallel line passing through the inferior pontine notch, thereby separating the pons from the medulla oblongata (see Figure 2). Using these anatomical boundaries, polygons were manually drawn to delineate the midbrain and pons, and the areas were then computed.

Polygons were used for area measurements, and ellipses for diameter measurements. For diameter measurements, ellipses were fitted to the segmented regions, excluding the pontine tegmentum in the pons and the collicular plate in the midbrain. Maximum midsagittal anteroposterior diameters were then derived from the minor axes of the fitted ellipses.

### 2.6. Deep Learning Model

Let *F* denote the 128 × 128 MSP ROI image. For automated segmentation, we train a CNN *U*_*θ*_ to output a probability mask: *P*≔U_*θ*_(*F*). Binary segmentation masks are obtained by thresholding *P*.

**Figure 3:**
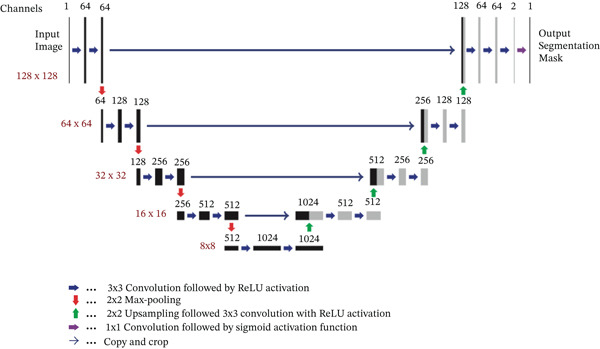
Used CNN architecture.

For the CNN architecture *U*_*θ*_, we use a standard U‐Net [34]. The CNN was implemented in Python using the Keras deep learning library. The U‐Net employs convolutions with filter sizes of 3 × 3 and 1 × 1, as well as max‐pooling and upsampling operations with a factor of 2. In total, the network contains 31,031,685 trainable parameters. An overview of the architecture is shown in Figure 3.

### 2.7. Loss Function

Training the network requires an objective function that measures how well the predicted segmentation mask matches the ground‐truth mask. Because the target structures (midbrain and pons) occupy only a small portion of the image, the data are strongly imbalanced. To address this, we use a combination of Dice loss and weighted binary cross‐entropy, which stabilizes optimization and ensures that small anatomical structures are not ignored (compare [35, 36]). The final loss is defined as the sum of Dice loss and weighted cross‐entropy loss:

LG,G∧≔LDiceG,G∧+LWCE G,G∧,

where

LDiceG,G∧≔1−2∑iGiG∧i∑iGi+∑iG∧iLLWCEG,G∧≔∑i∈I∑k=01,wi·Gi−k·logk−G∧iw≔log1+∑k=01,I·1IkIk+I·1CkCk.

To aid clarity, all variables used above are explicitly defined:

1. *I* : set of all pixel indices in the image
2. *G*[*i*] : ground‐truth label of pixel *i* (1 = structure, 0 = background)
3. $\overset{\wedge }{G}\left[i\right]$: predicted probability for pixel *i*
4. *I*_1_: set of all pixels belonging to the target structure
5. *C*_1_: set of contour pixels of the structure
6. |*A*| : number of pixels in a set |*A*|
7. 1_A_ (*i*): indicator function (1 if *i* ∈ *A*, 0 otherwise)
8. *w*[*i*]: class‐ and contour‐aware weighting factor for pixel *i*

Dice loss penalizes spatial mismatches between prediction and ground truth, ensuring high overlap and preserving structural shape while remaining robust to class imbalance. Weighted cross‐entropy emphasizes pixel‐wise accuracy for small target structures by assigning higher weights to minority‐class and boundary pixels, preventing trivial background‐only predictions. Together, these terms form a robust loss function tailored to the small, clinically relevant regions of the MSP.

### 2.8. Model Training

For each segmentation task (area or diameter; midbrain or pons), a separate model was trained. All models were based on data from 56 patients with multiple sclerosis (61% female, mean age 46.4 years), comprising a total of 136 MRI scans. The training dataset included 136 pairs of MRI scans and their corresponding binary segmentation masks. Networks were trained for 30 epochs with a batch size of 4. After each epoch, model performance was evaluated on the validation set using the Keras accuracy metric, and the weights achieving the best validation performance were retained as the final model.

To improve robustness, data augmentation was applied using the ImageDataGenerator module in Keras, including random horizontal and vertical shifts of −10 to 10 pixels, rotations of −15° to 15°, and zooms of 80%–120%.

### 2.9. Automated Planimetry

Automated planimetry was applied to the four trained neural networks, each targeting a specific task. For automated area measurements, the number of pixels in the binary segmentation masks produced by the networks was counted and multiplied by the pixel area. For diameter measurements, an ellipse was algorithmically fitted to the corresponding regions. First, the boundary points of the binary segmentation mask were selected, which do not form perfect ellipses. An ellipse was then calculated to best fit this set of boundary points in a least‐squares sense. The diameter was defined as the length of the minor axis of this fitted ellipse.

### 2.10. Evaluation Metrics

To quantitatively assess agreement between automated and manual segmentations, several complementary metrics were used to capture spatial overlap, area and diameter accuracy, and boundary distance:

1. Sørensen–Dice coefficient (DICE)
2. Area difference (AD)
3. Relative absolute area difference (RAAD)
4. Absolute diameter difference (ADD)
5. Relative absolute diameter difference (RADD)
6. Hausdorff distance (HAUS)

DICE measures spatial overlap between masks (1 = perfect agreement). AD and RAAD quantify absolute and relative differences in segmented area, while ADD and RADD evaluate differences in anatomically relevant diameters. HAUS assesses boundary accuracy by measuring the maximum contour deviation. Together, these metrics provide a comprehensive evaluation of geometric accuracy and segmentation reliability, which is essential for reproducible planimetric measurements.

Formally, these quantities are defined as

DICE≔2A∩BA+BAD≔A−B×pixel sizeRAAD≔2  A−B A+B×100ADD≔diamA−diamBRADD≔2diamA−diamBdiamA+diamBHAUS≔maxmaxa∈A da,B,maxb∈B db,A,

where *A* denotes the manual segmentation mask and *B* the automated segmentation mask, diam(·) is the diameter, and *d*(*x*, *Y*) denotes the Euclidean distance between a point *x* and a set *Y*.

### 2.11. Evaluation

To evaluate the methods, an independently selected third dataset of 54 images from 22 patients was used. Automated planimetric measurements were compared with manual measurements, which served as the gold standard. Both manual and automated segmentations were performed on the same MSP determined by the algorithm described in Subsection 2.4. All evaluation metrics from Subsection 2.10 were calculated, and each segmentation mask was visually inspected and approved by an independent expert. Correlation analysis was conducted, and robustness was further assessed using a second independent dataset. Data splitting was performed at the patient level to prevent data leakage between training, test, and evaluation sets.

## 3. Results

### 3.1. Area Measurements

We first evaluated area measurements of the midbrain and pons on the test set. Figure 4 illustrates the two best and two worst segmentation results for the trained U‐Net based on DICE scores for the pons and midbrain, respectively. The images in the last column of the lower panel show a tumor in the hypothalamus, representing a challenging out‐of‐distribution case. DICE scores for pons segmentations ranged from 0.984 (best) to 0.901 (worst), while midbrain segmentations ranged from 0.984 to 0.892. Even the segmentations with the lowest DICE scores still closely resemble the ground truth.

Figure 4Comparison between manual (lower row) and automatic (upper row) segmentations for the (a) best and (b) worst cases based on the pons (two left columns) and midbrain (two right columns) segmentation. Segmentations of the midbrain are marked in green, and segmentations of the pons are marked in blue. These examples show that automated segmentations closely follow expert contours even in difficult cases.

**Figure:**
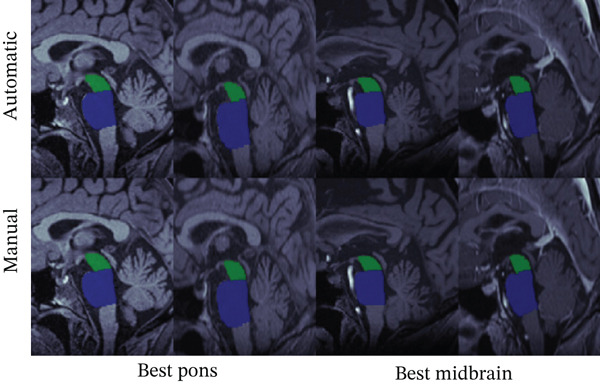
(a)

**Figure:**
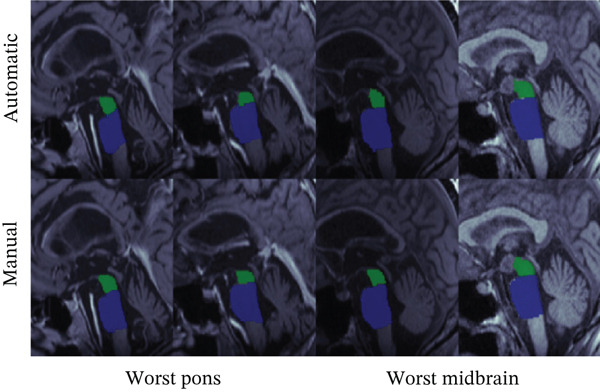
(b)

A quantitative performance analysis is presented in Table 3, where manual segmentations are used as the ground truth. For all accuracy measures, the mean, standard deviation (SD), and 95% confidence interval were calculated on the test data.

**Table 3:** Performance analysis of automated segmentation compared to manual segmentation used as ground truth on the test data. Statistics are provided for DICE, RAAD, and HAUS averaged over the MS test dataset including 54 images.

Ellipse metrics (*m* *e* *a* *n* ± *S* *D*)			
	DICE	RAAD	HAUS
Midbrain	0.941 ± 0.02	4.52 ± 3.64	1.24 ± 0.43
Pons	0.966 ± 0.011	3.02 ± 2.06	1.59 ± 0.64

### 3.2. Diameter Measurements

Next, we compare automated and manual ellipses and corresponding diameter measurements. As explained in Subsection 2.9, the calculation of the diameter is done in two consecutive steps consisting of CNN segmentation and ellipse fitting using the minor axis as the diameter. Each ellipse found by the algorithm was visually inspected by an expert. In general, the quality of the automatically found ellipses was viewed as high, meaning that no substantial misclassification was found. Figure 5 shows examples of the automatically found ellipses compared to manually fitted ellipses. Mean values and SD for *D* *I* *C* *E*, *A* *D* *D*, and *R* *A* *D* *D* are shown in Table 4.

Figure 5Comparison between manual (lower row) and automatic (upper row) segmentations for the (a) best and (b) worst cases based on the pons (two left columns) and midbrain (two right columns) segmentation. Segmentations of the midbrain are marked in green, and segmentations of the pons are marked in blue.

**Figure:**
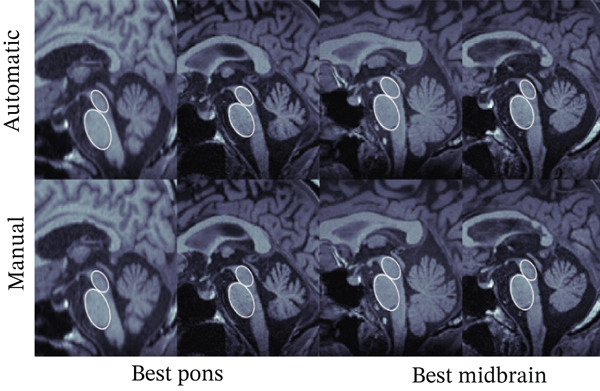
(a)

**Figure:**
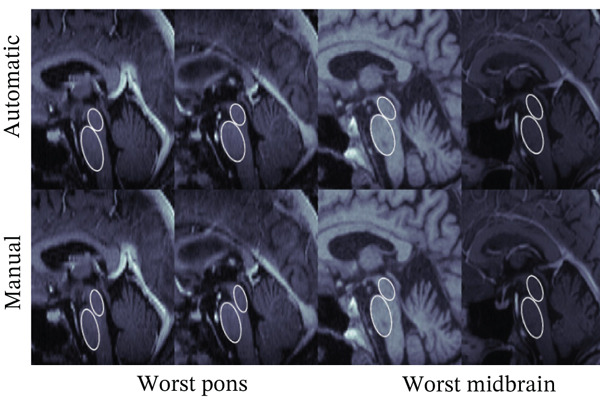
(b)

**Table 4:** Performance analysis for diameter measurements. Statistics are for the Sørensen–Dice coefficient (DICE), the absolute diameter difference (ADD), and the relative absolute diameter difference (RADD) taken over an evaluation dataset including 54 images.

Ellipse metrics (*m* *e* *a* *n* ± *S* *D*)			
	DICE	ADD	RADD
Midbrain	0.908 ± 0.046	0.400 ± 0.198	8.31 ± 4.86
Pons	0.925 ± 0.033	0.496 ± 0.368	5.90 ± 4.41

### 3.3. Regression and Correlation Analysis

We evaluated the agreement between manual and automated segmentations on the test data. Automated and manual area measurements for the midbrain and pons were highly correlated (*R* ^2^ = 0.878 and 0.886, respectively; *p* < 10^−25^), as shown in Figure 6a,b. Bland–Altman plots indicate a slight tendency for automated oversegmentation, with mean ADs of 4.21 mm^2^ (midbrain) and 9.33 mm^2^ (pons) and limits of agreement (mean ±1.96*σ*) corresponding to *σ* = 7.08 and 17.99 mm^2^, respectively.

Figure 6Regression analysis for (a) midbrain area and (b) pons area on the test data. The horizontal axis is for manual, and the vertical axis is for automated area values. Bland–Altman plots for (c) midbrain and (d) pons. The horizontal axis is the average, and the vertical axis is the difference between automated and manual area values. Strong correlations and tight Bland–Altman limits indicate minimal bias and support the use of automated planimetry for longitudinal monitoring.

**Figure:**
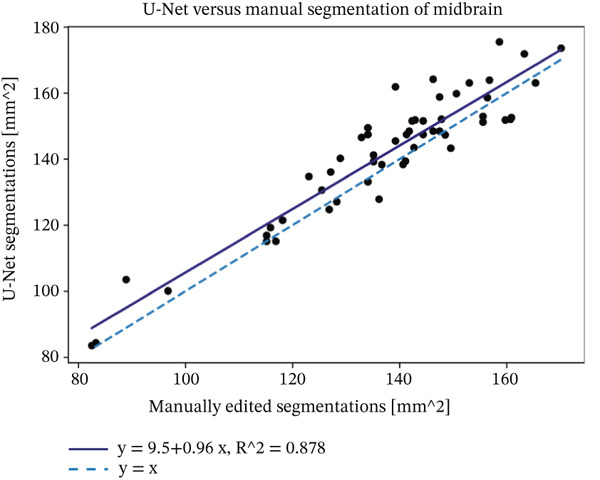
(a)

**Figure:**
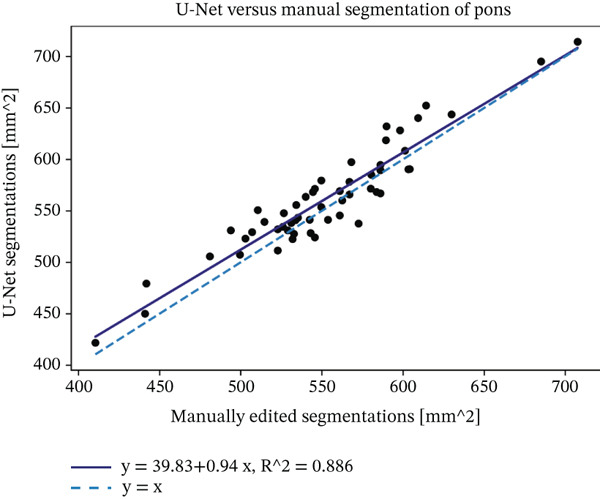
(b)

**Figure:**
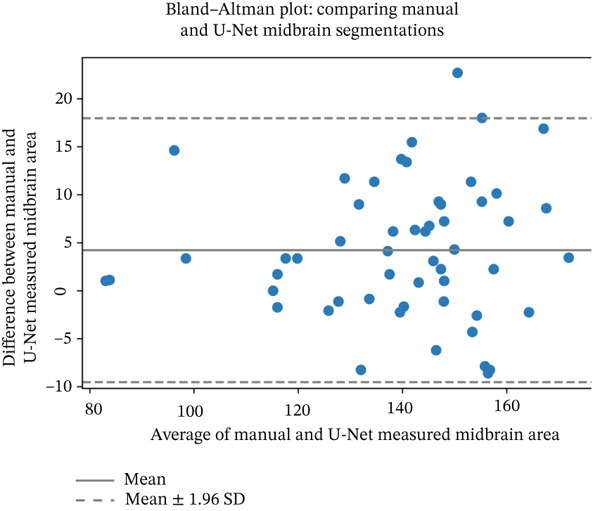
(c)

**Figure:**
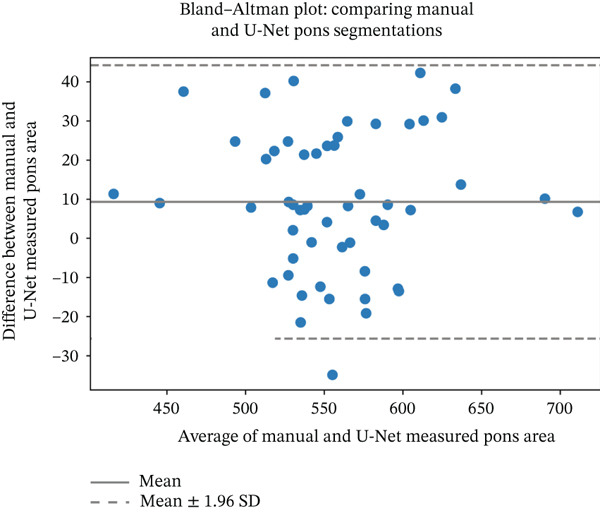
(d)

Ellipse‐derived diameters were similarly well correlated with manual measurements (*R* ^2^ = 0.780 for midbrain, *R* ^2^ = 0.794 for pons; *p* < 10^−18^), confirming that the automated approach accurately captures both area and diameter metrics (see Figure 7).

Figure 7Regression analysis for automatically and manually found diameters of (a) midbrain and (b) pons.

**Figure:**
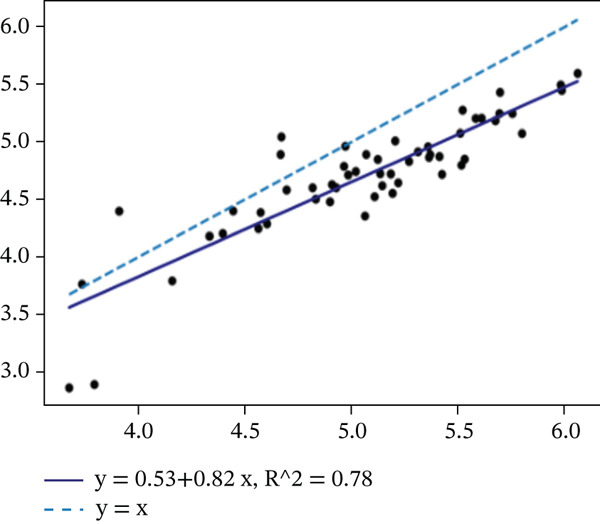
(a)

**Figure:**
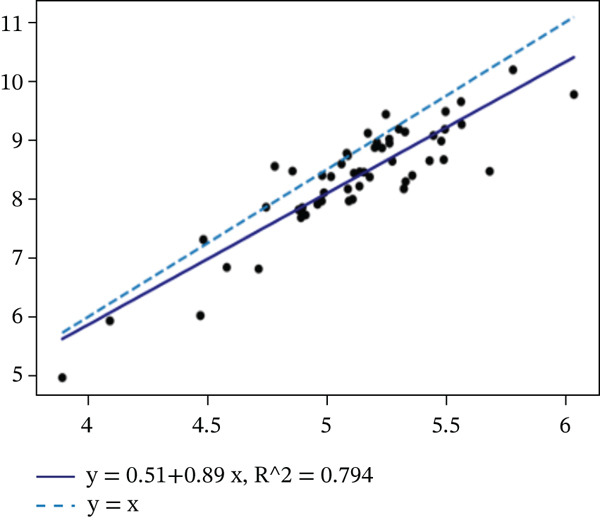
(b)

### 3.4. Robustness Against MRI Settings

The test cohort contains 13 MRI volumes where a contrast agent was used. Further, different pixel spacings and slice thicknesses were used during the MRI measurements. The big subgroups consist of images sized 128 × 256 with a slice thickness of 1.2 mm and images sized 176 × 256 with a slice thickness of 1 mm. The influence of these parameters is shown in Table 5. Student′s *t*‐test revealed that differences between results with and without contrast agent were not significant (*p* ≥ 0.5).

**Table 5:** Analysis comparing the use of contrast agent (CA), where Y stands for contrast agent and N for no contrast agent, and the influence of the image size *N*_*z*_ × 256 where *N*_*z*_ = 128 or *N*_*z*_ = 176. The symbol “#” denotes the number of test samples.

	Area midbrain (*m* *e* *a* *n* ± *S* *D*)			Area pons (*m* *e* *a* *n* ± *S* *D*)		
CA, *N*_*z*_, #	DICE	RAAD	HAUS	DICE	RAAD	HAUS
N, 128, 28	0.938 ± 0.019	5.20 ± 3.74	1.28 ± 0.470	0.967 ± 0.009	2.60 ± 1.83	1.48 ± 0.424
N, 176, 10	0.954 ± 0.017	2.23 ± 1.87	1.18 ± 0.335	0.973 ± 0.006	2.89 ± 1.52	1.57 ± 0.528
Y, 128, 11	0.933 ± 0.025	5.57 ± 4.30	1.30 ± 0.491	0.960 ± 0.013	4.07 ± 2.89	1.89 ± 0.99

On the other hand, when 176 × 256 MRI scans were compared to the 128 × 256 without a contrast agent, we received a *p* value of 0.02 for the DICE in the midbrain. The comparison to smaller sized images with contrast agent led to similar results *p* = 0.03. Further, with *p* values of 0.003 (without contrast agent) and 0.03 (with contrast agent), respectively, we received significant differences for the midbrain RAAD. The pons DICE led to *p* values of 0.05 (without contrast agent) and 0.01 (with contrast agent), respectively. On the other hand, there were no significant differences for the pons concerning the RAAD.

### 3.5. Robustness Against Noise

To further test the robustness of our framework, we evaluated its performance on MRI scans corrupted with noise. As it is well known that noise in MRI magnitude images follows a Rician distribution [37], experiments were performed for both Rician and Gaussian noise. To this end, noise was added at varying signal‐to‐noise ratios (SNRs) to simulate real‐world degradation. The segmentation performance was measured using Dice coefficients, and failure cases were identified as empty segmentation masks. Results show that the network maintains high accuracy at moderate noise levels, but performance begins to decline as the SNR drops below 15. Notably, we observed a sharp threshold effect: The segmentation either works well or fails completely beyond a certain noise level. However, this threshold varies from image to image, suggesting that each scan has an individual noise tolerance limit (see Figure 8).

Figure 8(a) Mean Dice coefficients of segmented images corrupted with Gaussian and Rician noise for different SNRs and (b) rate of zero outputs from the network for the two noise models at different SNRs.

**Figure:**
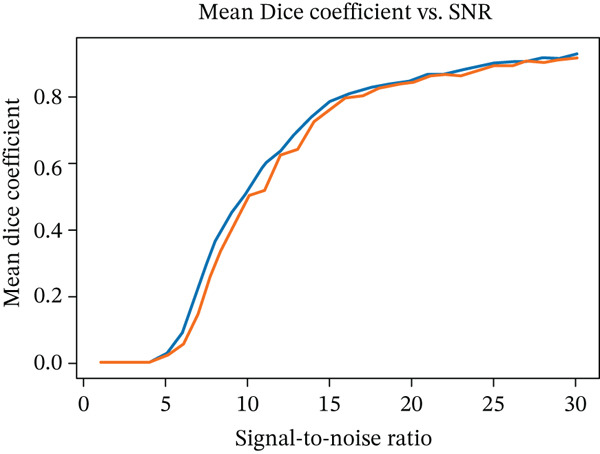
(a)

**Figure:**
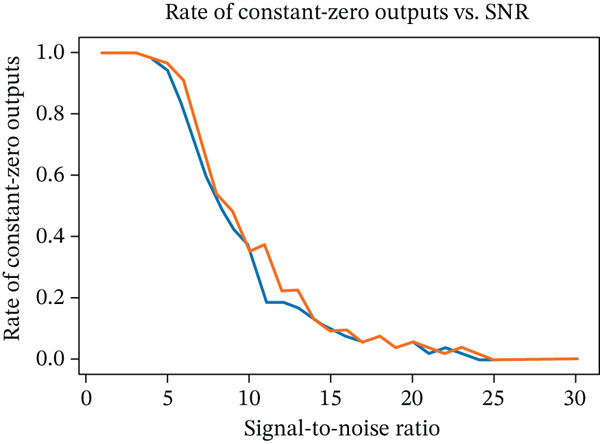
(b)

### 3.6. Test on Healthy Control Group

To test the robustness of our algorithm with respect to the MRI class, we also examined the performance on a test set of 19 healthy controls. The results are shown in Table 6. One notices that error metrics are comparable to the error metrics on the MS patients, even though neither of the networks has been trained on healthy patients. This further supports the robustness of our approach.

**Table 6:** Means and SD of the different error measures on a sample of 19 healthy patients.

	Areas (*m* *e* *a* *n* ± *S* *D*)			Ellipses (*m* *e* *a* *n* ± *S* *D*)		
	DICE	RAAD	HAUS	DICE	RAAD	HAUS
Midbrain	0.929 ± 0.018	6.06 ± 4.78	1.12 ± 0.30	0.920 ± 0.030	0.206 ± 0.151	4.03 ± 2.95
Pons	0.961 ± 0.019	2.89 ± 3.07	1.51 ± 0.70	0.934 ± 0.025	0.525 ± 0.345	6.64 ± 4.33

### 3.7. Comparison to Manual Rating

Table 7 presents a comparison of the automated network segmentations with two independent raters. The results from two raters, one with professional radiological skills and the other a student, were compared. They also had varying levels of experience with MR planimetric measurement performance. Additionally, the two raters are compared to each other. One can observe that the CNN is able to maintain correlations between the two expert raters. This comparison demonstrates that 2D measurements remain unaffected by user experience.

**Table 7:** Means and SD comparing the different measurements (two independent raters and the proposed algorithm). The best values are marked in bold.

	CNN vs. R1	CNN vs. R2	R1 vs. R2
Area DICE (mean ± SD)			
MB	0.902 ± 0.028	0.898 ± 0.033	0.900 ± 0.033
Pons	0.962 ± 0.010	0.954 ± 0.011	0.953 ± 0.012
Area AD (mean ± SD)			
MB	7.95 ± 6.60	12.23 ± 8.19	11.4 ± 8.03
Pons	19.7 ± 14.0	31.6 ± 19.9	44.3 ± 15.2
Ellipse DICE (mean ± SD)			
MB	0.918 ± 0.045	0.924 ± 0.053	0.920 ± 0.035
Pons	0.928 ± 0.029	0.888 ± 0.042	0.928 ± 0.038
Ellipse ADD (mean ± SD)			
MB	0.185 ± 0.292	0.226 ± 0.364	0.025 ± 0.212
Pons	0.240 ± 0.401	0.751 ± 0.413	0.598 ± 0.370

### 3.8. Out of Distribution Cases

We analyzed patients whose midsagittal structures deviated substantially from those of all cases in the training and evaluation datasets. The first case involves a mass of unknown origin in the mammillary bodies (Figure 9a, marked with an arrow). Despite this anomaly, the U‐Net successfully segments the midbrain, likely benefiting from its learned shape priors and subtle intensity differences. The second case, which also appears only in the test dataset, involves an elongated basilar artery pressing against and slightly deforming the pons (Figure 9b, marked with an arrow). In this instance, the network misclassifies part of the artery as pontine tissue, likely due to minimal intensity contrast and the largely preserved geometric structure.

Figure 9Two out‐of‐distribution cases are shown: (a) a tumor in the mammillary bodies (marked by an arrow) and (b) a basilar artery displaced against the pons (marked by an arrow). Each example includes the MRI scan (left), the manual segmentation (middle), and the network prediction (right).

**Figure:**
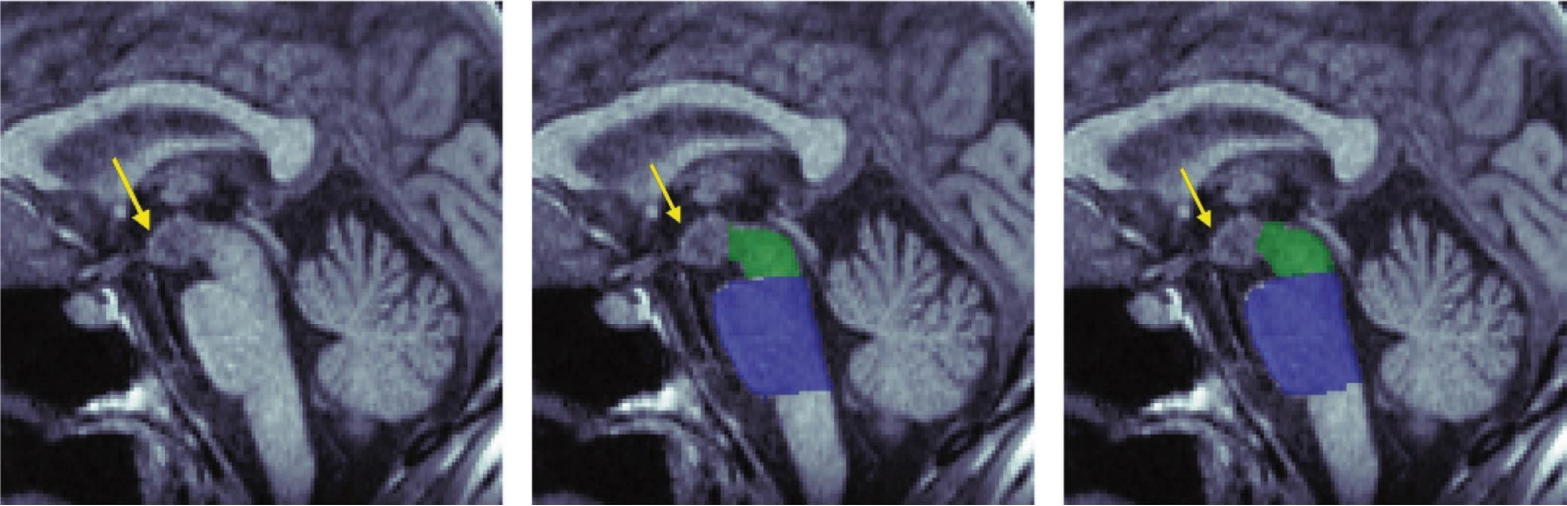
(a)

**Figure:**
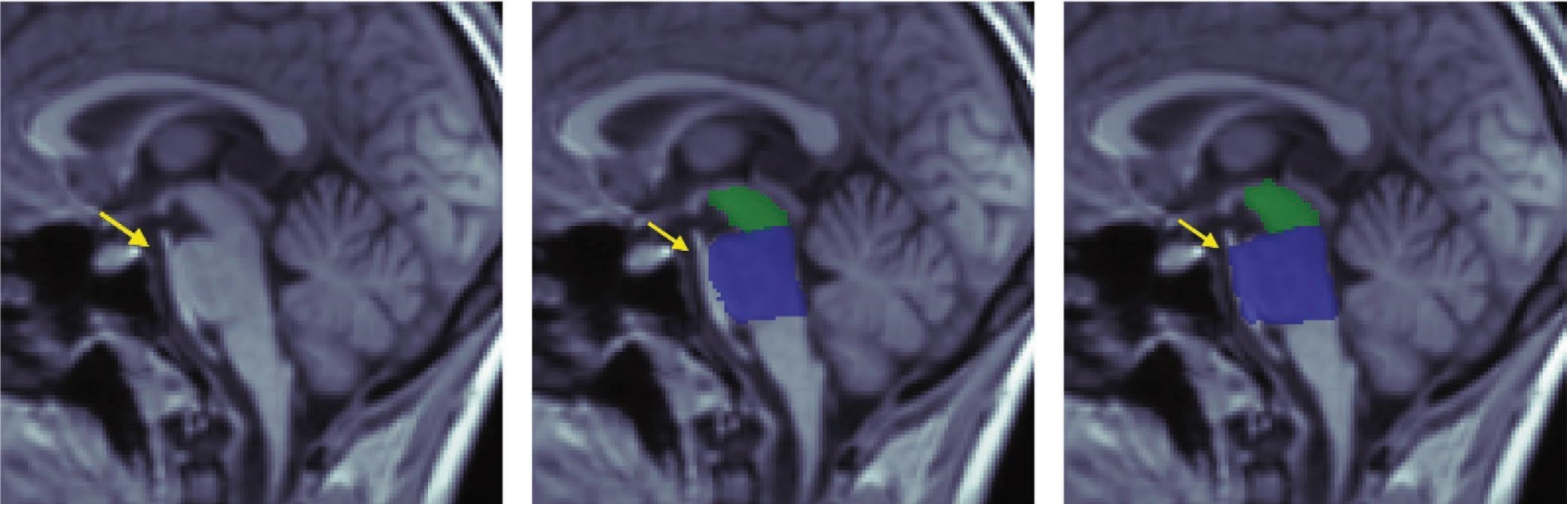
(b)

We emphasize that the training data did not contain any cases in which the basilar artery was displaced against the pons. We therefore conjecture that expanding the training dataset to include such anatomical variations would help overcome this limitation. Another complementary strategy may involve targeted data augmentation tailored to this specific challenging scenario.

## 4. Discussion

MRI planimetry is an established tool for assessing brain atrophy in MS and is closely linked to disease progression and treatment response [38]. Brainstem atrophy, in particular, has emerged as a significant contributor to disability progression and neurological decline and is a reliable predictor of future physical and cognitive impairment [39, 40]. In this work, we present a deep learning framework that automates MRI planimetry by integrating an MSP detection algorithm with a neural network for segmentation and measurement, aiming to mitigate the limitations of manual assessment and scanner‐related variability [41–44]. Because planimetric segmentation is performed on a single MSP slice, the pipeline is fast and reduces analysis time by automating plane selection. Processing all four regions of interest (ellipses and polygons for pons and midbrain) required 0.45 s per patient on a standard PC with 8 GB RAM, compared with 106.7 s for manual segmentation [9].

The method was developed using a large database spanning all clinical MS phenotypes (clinically isolated syndrome, primary progressive MS, secondary progressive MS, and relapsing–remitting MS) and evaluated on an independent clinical cohort acquired on different MRI systems as part of routine care. These results support the feasibility and generalizability of automated infratentorial planimetry in real‐world settings. U‐Net‐based segmentations showed high agreement with manual labels, with mean DICEs of 0.966 (pons) and 0.941 (midbrain) on the test set [45]. Boundaries between brainstem substructures (pons, midbrain, and medulla) were the most challenging, yet automated contours typically deviated by only one‐to‐two‐pixel slices from manual delineations. This segmentation accuracy translated into strong concordance of planimetric areas: The relative absolute difference (RAD) was 3.02% for pons and 4.52% for midbrain, and Dice coefficients exceeded 0.89 for both structures across all test cases.

Ellipse‐based measurements likewise showed high spatial overlap between automated and manual fits (mean Dice: 0.925 for pons; 0.908 for midbrain). Diameter estimates were accurate (mean RAD: 5.9% for pons; 8.31% for midbrain) and strongly correlated with manual measurements. Consistent with prior observations, automated diameters were slightly smaller than manual ones in most cases (85% of pons; 92% of midbrain), but this small negative bias did not materially affect the correlations. This likely reflects mild underestimation in the ellipse‐fitting step, which can yield slightly shorter diameters even when the underlying automatic segmentations delineate larger areas. Alternative fitting strategies—such as minimizing the sum of squared geometric distances to boundary points rather than using algebraic fits—could mitigate this bias in future versions. With respect to clinical thresholds, this bias did not lead to threshold‐based reclassifications in our sample; however, measurements very close to a cutoff may warrant manual confirmation or a small calibration offset. Overall, we consider the higher consistency of the automatic measurements more important than the minor systematic underestimation, which can be accommodated in clinical decision‐making.

Robustness analyses indicated that performance remained stable down to an SNR of 15, well within the typical clinical range of 20–50. Generalization to healthy subjects—despite training exclusively on MS data—yielded Dice coefficients comparable to those in the MS test set. Contrast administration had no measurable effect on segmentation, whereas slice thickness (axial resolution) was a key determinant of accuracy, as expected.

Automated planimetry reduces observer dependence and improves reproducibility compared with manual assessments, which are sensitive to rater interpretation, scanner type, and acquisition parameters [42, 46]. This stability is particularly important for longitudinal monitoring, where small differences can change the clinical interpretation of progression and inform treatment decisions. More reliable tracking of brainstem atrophy could support earlier identification of patients at risk of disability worsening (e.g., in relation to EDSS) and guide escalation when disease‐modifying therapy (DMT) fails to slow neurodegeneration [38, 47]. Standardized, automated measurements may also reduce intersite variability in multicenter trials, strengthening study power and interpretability [41].

Limitations and future work include the following: (1) While segmentation accuracy was high, the method does not enforce parallel boundaries between pons and midbrain; this could be addressed via alternative loss functions, postprocessing constraints, or geometric regularizers. (2) The ellipse‐fitting routine showed a slight negative diameter bias relative to manual measurements, in contrast to the small positive bias in segmented areas. Approaches minimizing geometric distance metrics (e.g., [48]) may mitigate this in future versions. (3) Although manual planimetry correlates with clinical outcomes, analogous analyses for automated measures remain to be established; future studies should test associations with disability and treatment response using longitudinal cohorts.

We observed incidental findings in our cohort, including three cases of basilar artery dolichoectasia mildly indenting the brainstem and one mammillary body tumor. The tumor did not affect segmentation; dolichoectasia led to localized misclassification. Given its prevalence in older, largely male, hypertensive populations and the younger, predominantly female demographic of MS, this represents a limited and incidental constraint on performance. The mammillary body lesion (likely a hamartoma) is extremely rare and does not alter the overall conclusions [49]. Beyond MS, automated planimetry may extend to disorders with brainstem involvement, such as PSP and MSA, where brainstem atrophy is diagnostically informative [28, 29, 50]. Future work should integrate additional biomarkers (e.g., spinal cord atrophy and functional MRI) and correlate automated metrics with standardized disability scales (e.g., EDSS) to refine clinical utility [51].

For clinical adoption, integration into radiology workflows is essential. The system can export DICOM Segmentation and Structured Reports for automatic ingestion into PACS/reporting platforms (including via DICOMweb), enabling overlays and quantitative metrics at the radiologist′s workstation. Deployment models include on‐premises installation, vendor‐neutral microservices within PACS/RIS (via DICOM SR and HL7), or cloud‐based inference for multisite scalability. Prior implementation studies show that structured report transfer of AI‐derived measurements improves efficiency, reduces transcription errors, and enables sub‐5‐min turnaround times at scale [52–56].

In summary, the proposed framework delivers fast, robust, and reproducible brainstem planimetry across scanners and acquisition settings. By reducing observer dependence and standardizing measurements, it has the potential to improve longitudinal monitoring, support treatment decisions, and accelerate the translational use of quantitative MRI biomarkers in MS care.

## 5. Summary and Outlook

In this paper, we present a U‐Net‐based segmentation approach to calculate the areas and diameters of the midbrain and pons on the MSP. Comparing the U‐Net‐based to manual segmentations yielded high Sørensen–Dice coefficients, indicating high accuracy in image segmentation. The automatically and manually measured areas of the pons and midbrain were highly correlated. Similarly, the spatial overlap between manually and automatically drawn ellipses was very high, leading to very accurate predictions of the diameters of the midbrain and pons. Our study demonstrates the feasibility of automated MRI planimetric segmentations of infratentorial structures in a clinical setting using acquired MRI scans from a large cohort of MS patients. The algorithms were successfully tested on an independent cohort of patients recruited from an MS clinic and scanned using different MRI systems as part of their routine diagnostic workup. Evaluating the algorithm on a larger cohort of MS patients and healthy controls is an important direction for future research. Our results show that automatic MRI planimetry in patients with early to moderately advanced MS, recruited in a clinical setting, works stably, regardless of variability in scanners and acquisition parameters. The implementation of the deep learning framework into clinical practice may play an important role in reliably predicting disease progression and response to treatment in patients with MS.

We employ a supervised deep learning strategy based on CNNs, which remain a well‐established and experimentally powerful technique for medical image segmentation. At the time this study was conducted, CNN‐based methods were considered state‐of‐the‐art for this task. Our architecture is based on a standard CNN design (a U‐Net variant). We combine Dice loss and weighted binary cross‐entropy to address class imbalance, a common challenge in medical segmentation tasks. To the best of our knowledge, this paper is the first to present an automated approach to MRI planimetry. Consequently, a direct comparison with previous work is not possible. Future work will focus on integrating more recent segmentation architectures into our framework, potentially replacing the current U‐Net backbone. Promising alternatives include nnU‐Net [49], the transformer‐based TransBTS [50], fine‐tuned versions of the Segment Anything Model (SAM) for medical data [51], and foundation models developed specifically for medical imaging tasks [52]. Table 8 presents an overview of the U‐Net and these recent models, summarizing their main features, advantages, and limitations.

**Table 8:** Overview of possible segmentation architectures besides the proposed U‐Net.

Architecture	Main feature	Pros	Cons
U‐Net	CNN‐based encoder–decoder with skip connections	Simple, efficient, well‐established in medical imaging	May underperform on complex shapes or low‐contrast data
nnU‐Net [49]	Self‐configuring U‐Net‐based pipeline	No manual tuning needed; top performer in many benchmarks	Requires high computational resources
TransBTS [50]	Transformer‐augmented 3D segmentation	Captures long‐range dependencies; better global context	More complex training; slower inference
SAM (fine‐tuned) [51]	Pretrained on vast general datasets; adaptable	Zero‐shot/few‐shot capability; strong generalization	Requires fine‐tuning for medical data; not domain‐specific
MedSAM [52]	Foundation model for medical segmentation	Optimized for medical images; transferable across tasks	Still under validation; limited fine‐grained control

Future work may incorporate advanced loss functions that integrate geometric priors, such as boundary‐aware or surface‐based losses, to reduce segmentation drift at structure interfaces. Adding explicit anatomical constraints (e.g., enforcing smoothness, convexity, or topology preservation) could further enhance robustness. In addition, exploring transformer‐enhanced architectures such as TransBTS, Swin‐Unet, or foundation model–based approaches like MedSAM may improve generalization, particularly for out‐of‐distribution cases. These architectures offer stronger global feature modeling and may reduce reliance on high spatial resolution. To ensure clinical scalability, future studies should also validate the pipeline on multicenter datasets with diverse scanner types, acquisition protocols, and field strengths, allowing assessment of how well the method transfers to real‐world imaging environments.

## Author Contributions

S.M.: funding acquisition, conceptualization, supervision, methodology, investigation, and writing—original draft; D.S.: funding acquisition, conceptualization, methodology, supervision, and writing—review and editing; M.S.: methodology, software development, experimental validation, formal analysis, statistical analysis, and writing—original draft; M.T.: methodology, software development, experimental validation, formal analysis, statistical analysis, and writing—original draft; F.D.: data curation and writing—review; M.H.: conceptualization, methodology, supervision, resources, and writing—review and editing (major restructuring); E.R.G.: conceptualization, methodology, supervision, resources, and writing—review and editing.

## Funding

This work was funded by Medizinische Universität Innsbruck (10.13039/501100009980) and Universität Innsbruck (10.13039/501100012163). Open Access funding provided by Universitat Innsbruck/KEMÖ

## Conflicts of Interest

The authors declare no conflicts of interest.

## Data Availability

Research data are not shared.
